# Supplementary material for: Gut Microbiota and Metabolome Changes in Three Pulmonary Hypertension Rat Models
Source: Microorganisms. 2023 Feb 13;11(2):472. doi: 10.3390/microorganisms11020472 (PMC9959815; doi:10.3390/microorganisms11020472)
Supplement: Supplementary file 1 [file microorganisms-11-00472-s001.zip › Figure S1-S5 and Table S1.pdf]

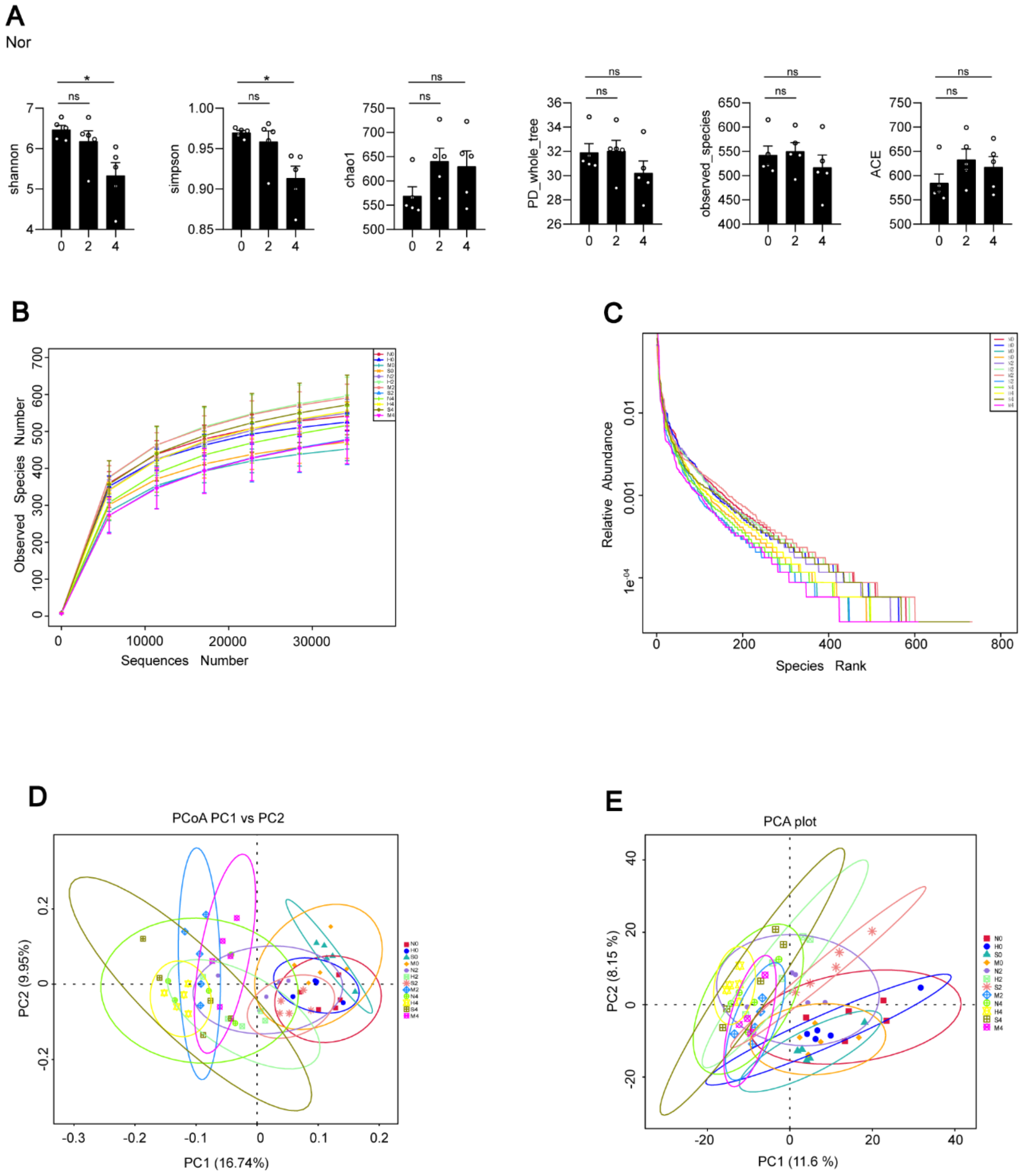

Supplementary Figure S1. The intestinal microbiota structure of Nor-, Hyp-, HySu-, and MCT-induced PH rats. Feces were collected from rats at weeks 0, 2, and 4. **(A)** The α diversity and β diversity of the gut microbiota in normoxic rats as measured by the Shannon, Simpson, Chao, PD whole tree, observed species indices and ACE. Chao1 estimates the number of species, whereas Shannon estimates the effective number of species. The results are presented as the mean ± SEM of 5 animals. *$P < 0.05$, ns: no significance. one-way ANOVA followed by Student-Newman-Keuls multiple comparison test was used for Shannon, Simpson, and observed species indices. The Kruskal–Wallis test followed by the Dunnett post hoc test were used for Chao, PD whole tree, and ACE. **(B)** Rarefaction curves for the observed species in the gut microbiota from Nor-, Hyp-, HySu-, and MCT-induced rats (n=5). **(C)** Rank abundance

curves. **(D, E)** Analysis of β diversity of gut microbiota in rats. Weighted UniFrac principal coordinates analysis (PCoA) **(D)** and principal component analysis (PCA) **(E)** of gut microbiota based on the OTU data of each sample of 12 group. Each point represents a sample. Nor (N): normoxia group; Hyp (H): hypoxia-induced group; HySu (S): hypoxia/Sugen 5416-induced group; MCT (M): MCT-induced group. 0, 2, and 4 represent the week of treatment.

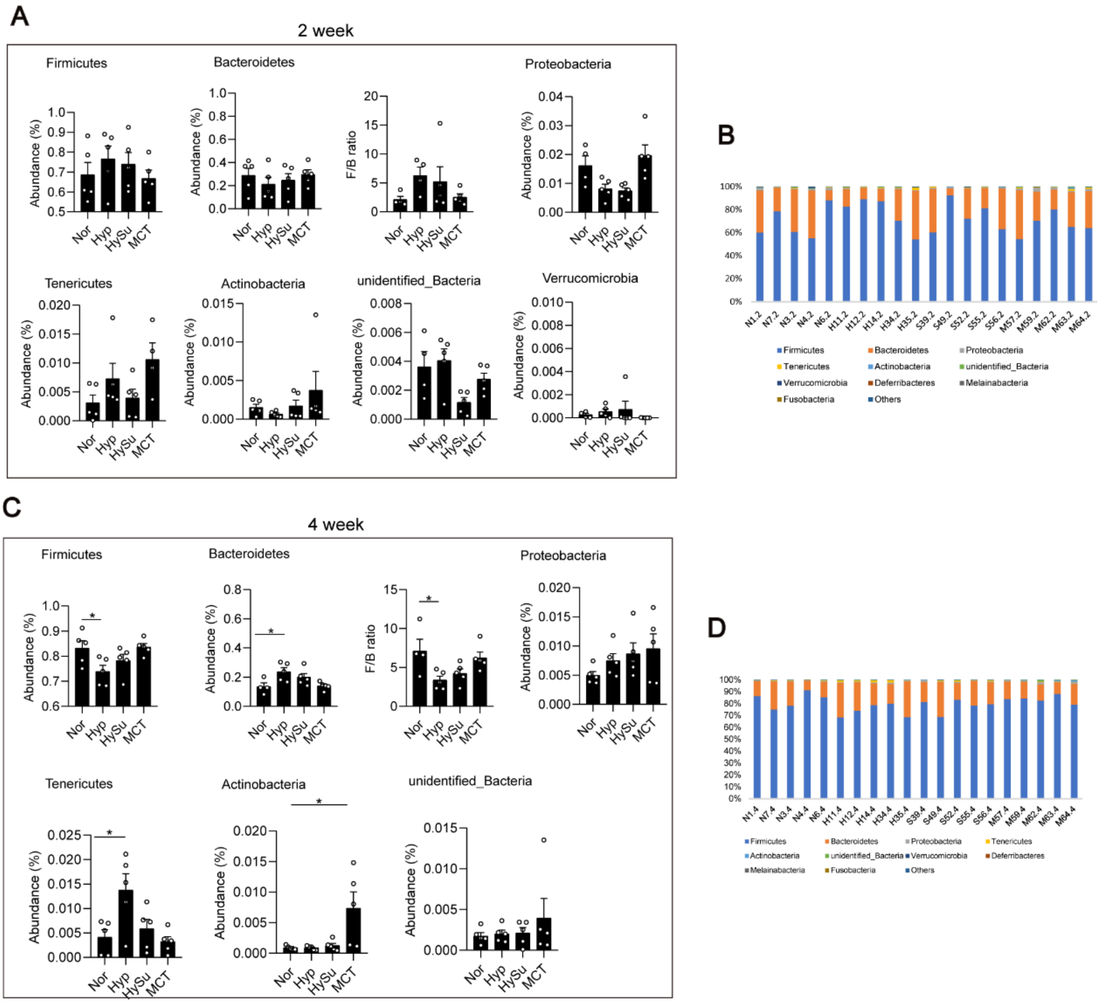

Supplementary Figure S2. Fecal microbial composition at the phylum level. **(A-D)** Changes in Firmicutes, Bacteroidetes, Proteobacteria, unidentified bacteria, Melainabateria and the ratio of Firmicutes/Bacteroidetes (n = 4-5 per group). Data are presented as the mean ± SEM. **P* < 0.05, ns: no significance. one-way ANOVA followed by Student-Newman-Keuls multiple comparison test for parametric distributions. The Kruskal–Wallis test followed by the Dunnett post hoc test were used for non-parametric distributions. N1, N3, N4, N6, and N7 represent the codes of rats in the normoxia group (Nor). H11, H12, H14, H34, and H35 represent the codes of rats in the Hyp-induced group (Hyp). S39, S49, S52, S55, and S56 represent the codes of rats in the HySu-induced group (HySu). M57, M59, M62, M63, and M64 represent the codes of rats in the MCT-induced group (MCT). 0, 2, and 4 represent the week of

treatment.

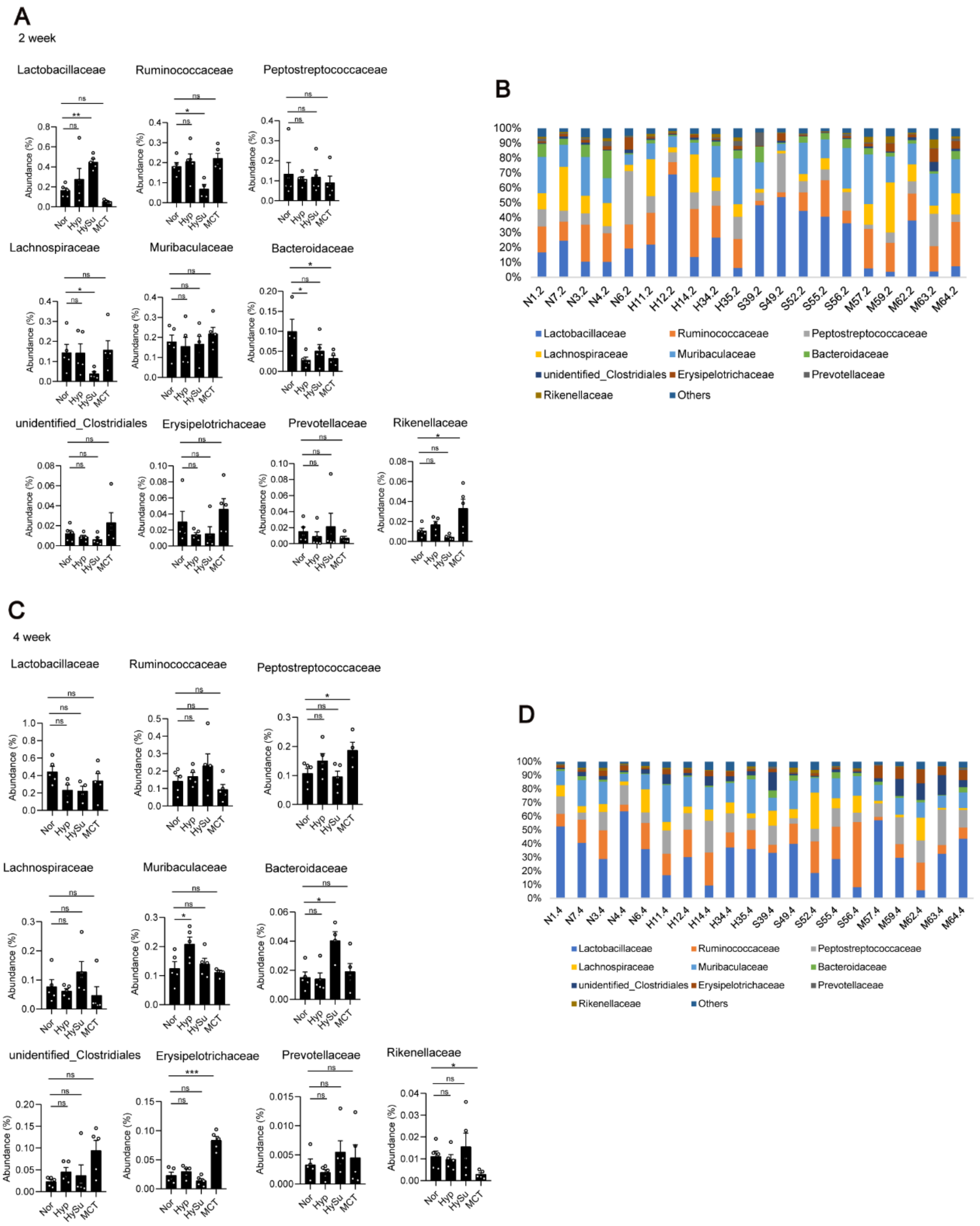

Supplementary Figure S3. Fecal microbial composition at the family level. (A-D) The most abundant taxa changes at the family level (n = 4-5 per group). Data are presented as the mean ± SEM. **P* < 0.05, ***P* < 0.01, ****P* < 0.001, ns: no significance. one-way ANOVA followed by Student-Newman-Keuls multiple comparison test for parametric

distributions. The Kruskal–Wallis test followed by the Dunnett post hoc test were used for non-parametric distributions. N1, N3, N4, N6, and N7 represent the codes of rats in the normoxia group (Nor). H11, H12, H14, H34, and H35 represent the codes of rats in the Hyp-induced group (Hyp). S39, S49, S52, S55, and S56 represent the codes of rats in the HySu-induced group (HySu). M57, M59, M62, M63, and M64 represent the codes of rats in the MCT-induced group (MCT). 0, 2, and 4 represent the week of treatment.

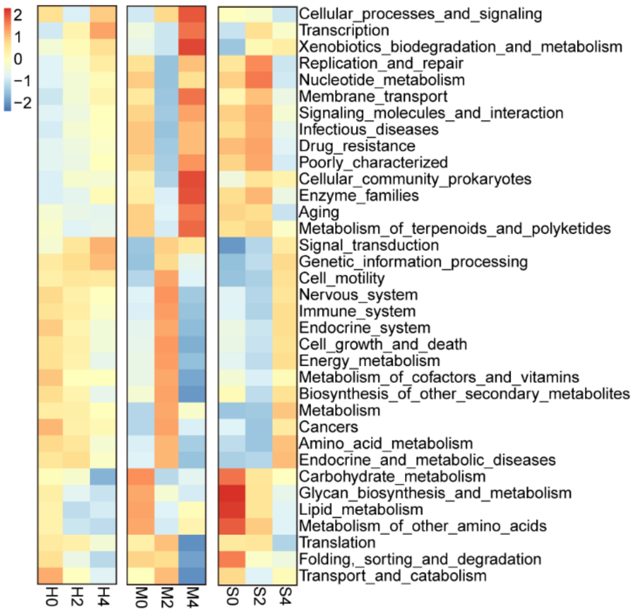

Supplementary Figure S4. Kyoto Encyclopedia of Genes and Genomes (KEGG) pathway analysis at level 2. Statistically significant KEGG pathways of the hypoxia-induced, hypoxia/Sugen 5416-induced and MCT-induced groups were determined using STAMP software, and a LEfSe (LDA score > 2) of significant KEGG pathways was performed. H: hypoxia-induced group; S: hypoxia/Sugen 5416-induced group; M: MCT-induced group; 0, 2, and 4 represent the week of treatment.

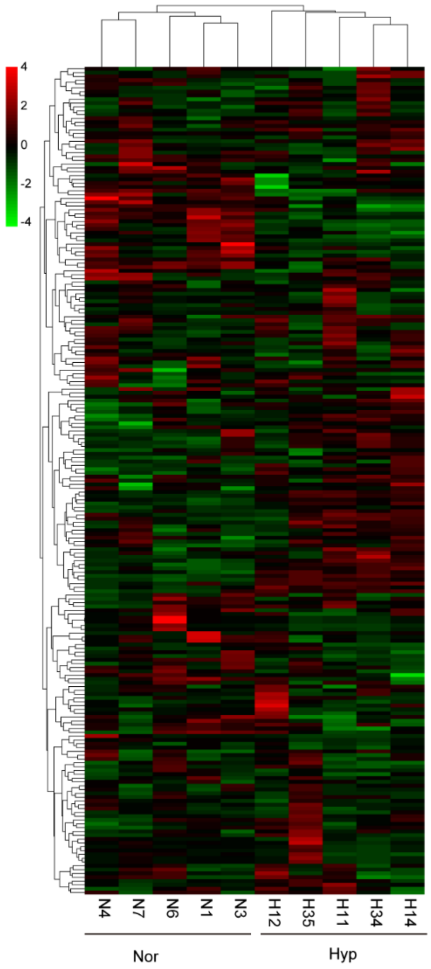

Supplementary Figure S5. Heatmap analysis of differential metabolites between the Nor and Hyp groups. N1, N3, N4, N6, and N7 represent the codes of rats in the normoxia group (Nor). H11, H12, H14, H34, and H35 represent the codes of rats in the Hyp-induced group (Hyp).

Supplementary Table S1. Number of reads and operational taxonomic units and the good coverage estimation for each sample from the pyrosequencing analysis.

#Sample_name	Raw_reads (#)	Clean_Reads (#)	Base(nt)	AvgLen(nt)	Q20	GC%	Effective%
N1.0	87698	80213	32881515	409	75.54	52.29	91.47
N7.0	84161	80177	33044666	412	72.11	52.44	95.27
N3.0	87208	80109	32599389	406	75.01	52.93	91.86
N4.0	86751	80142	32900001	410	73.98	52.4	92.38
N6.0	83152	80116	32404145	404	74.9	52.19	96.35
H11.0	83146	80065	33423708	417	71.82	51.67	96.29
H12.0	84440	80154	32712042	408	74.39	51.74	94.92
H14.0	85240	80124	32457535	405	72.75	52.65	94
H34.0	86528	80213	32564345	405	75.06	52.44	92.7
H35.0	88861	80110	32583688	406	75.11	52.26	90.15
M57.0	83440	80082	32961259	411	72.1	52.21	95.98
M59.0	83578	80119	33568313	418	71.89	51.66	95.86
M62.0	84612	80025	32870260	410	75.71	51.42	94.58
M63.0	87682	80234	33532017	417	71.1	51.58	91.51
M64.0	86017	80084	33265284	415	74.06	51.3	93.1
S39.0	69300	64471	26580812	412	72.01	52.19	93.03
S49.0	73109	67015	27813659	415	71.59	51.69	91.66
S52.0	89049	80234	33543422	418	73.67	51.41	90.1
S55.0	86604	80175	33639065	419	83.01	51.86	92.58
S56.0	86060	80086	33478123	418	83.05	52.11	93.06
N1.2	83784	80248	33326010	415	81.89	52.68	95.78
N7.2	87746	80175	33194636	414	80.71	52.86	91.37
N3.2	84992	80089	32953330	411	82.36	52.55	94.23
N4.2	84905	80093	33181212	414	80.51	51.96	94.33
N6.2	86086	80055	32937516	411	81.92	52.71	92.99
H11.2	87769	80093	32881642	410	81.58	52.86	91.25
H12.2	82763	80137	33830512	422	80.78	51.52	96.83
H14.2	83701	80192	32907873	410	81.39	52.7	95.81

H34.2	88797	80122	33234715	414	82.37	52.66	90.23
H35.2	82237	80158	33047645	412	80.74	52.87	97.47
M57.2	83266	80076	33045394	412	81.34	53.16	96.17
M59.2	83415	80202	32778739	408	79.8	53.56	96.15
M62.2	86462	80075	33307278	415	82.87	52.44	92.61
M63.2	86303	80023	33034422	412	80.62	52.94	92.72
M64.2	82174	80032	32898895	411	82.96	52.84	97.39
S39.2	84424	80224	33892158	422	81.95	51.86	95.03
S49.2	84691	80134	33684508	420	80.9	52.2	94.62
S52.2	84686	80170	33455203	417	82.77	52.17	94.67
S55.2	84876	80324	33349695	415	82.89	52.18	94.64
S56.2	82581	80042	33531436	418	81.6	52.71	96.93
N1.4	83254	80134	33618999	419	80.66	52.53	96.25
N7.4	86914	80096	33551768	418	81.18	52.45	92.16
N3.4	84566	80053	33090205	413	81.81	52.16	94.66
N4.4	84404	80096	33673849	420	82.05	52.12	94.9
N6.4	82364	80024	33274486	415	81.05	52.39	97.16
H11.4	85204	80119	33191783	414	81.94	52.96	94.03
H12.4	86571	80133	33266167	415	82.43	52.41	92.56
H14.4	87170	80150	32892940	410	82.03	52.86	91.95
H34.4	88717	80060	33277595	415	82.04	52.54	90.24
H35.4	81706	80043	33531468	418	80.65	52.4	97.96
S39.4	87926	80308	33436633	416	81.91	52.57	91.34
S49.4	85230	80102	33536221	418	82.36	52.29	93.98
S52.4	87921	80190	32785883	408	82	53.36	91.21
S55.4	86523	80030	33041848	412	82.18	52.47	92.5
S56.4	88141	80209	32732908	408	81.17	53.25	91
M57.4	74428	72483	30575737	421	86.89	52.09	97.39
M59.4	85191	80048	33372336	416	83.3	52.6	93.96
M62.4	84158	80070	32810591	409	85.46	52.96	95.14
M63.4	85678	80095	33411923	417	82.41	52.46	93.48
M64.4	83754	80273	33661411	419	84.4	51.89	95.84
